# Direct detection and identification of periprosthetic joint infection pathogens by metagenomic next-generation sequencing

**DOI:** 10.1038/s41598-023-35215-3

**Published:** 2023-05-16

**Authors:** Linjie Hao, Pengfei Wen, Wei Song, Binfei Zhang, Yanjie Wu, Yumin Zhang, Tao Ma, Yusheng Qiu

**Affiliations:** 1 Department of Orthopedics, The First Affiliated Hospital of Xi’an Jiaotong University, 277 West Yanta Road, Xi’an, 710061 Shaanxi China; 2 Department of Joint Surgery, Honghui Hospital, Xi’an Jiaotong University, 555 East Youyi Road, Xi’an, 710000 Shaanxi China

**Keywords:** Biological techniques, Medical research

## Abstract

This study assessed the application of metagenomic next-generation sequencing in pathogen detection of periprosthetic joint infections. A total of 95 cases who previously had undergone hip and knee replacement undergoing revision from January 2018 to January 2021 were included in this study. Specimens of synovial fluid and deep-tissue were collected for culture and metagenomic next-generation sequencing, and patients were retrospectively categorized as infected or aseptic using the Musculoskeletal Infection Society criteria after revision surgery. The sensitivity, specificity, positive and negative predictive values were compared. A total of 36 cases had positive culture results and 59 cases had positive metagenomic next-generation sequencing results. Culture was positive in 34 infected cases (58.6%) and 2 aseptic cases (5.4%). Metagenomic next-generation sequencing was positive in 55 infected cases (94.8%) and 4 aseptic cases (10.8%). Five cases diagnosed with infection had other potential pathogens detected by metagenomic next-generation sequencing. Among the 24 culture-negative periprosthetic joint infections, metagenomic next-generation sequencing was able to identify potential pathogens in 21 cases (87.5%). From sampling to reporting, the average time needed for culture was 5.2 (95% CI 3.1–7.3) days, while that for metagenomic next-generation sequencing was 1.3 (95% CI 0.9–1.7) days. Metagenomic next-generation sequencing is more advantageous in pathogen detection of periprosthetic joint infection after total joint replacement, especially in patients with multiple infections or negative culture results.

## Introduction

Total joint replacement (TJR) is an effective treatment for end-stage bone diseases, such as osteoarthritis, rheumatoid arthritis, and traumatic arthritis, while periprosthetic joint infection (PJI) is one of the most severe complications after the replacement^1^, which brings heavy burden on both patients and the medical system and severely affects the patients’ quality of life^2^. The incidence of PJI after a primary total joint replacement for knee or hip is about 0.7–2%^3,4^, while the incidence of PJI after revision operation is about 5.6–35%^5–7^. Although with increasing advances in surgical techniques and materials science, the incidence of PJI is decreasing, due to the large number of patients undergoing total joint replacement, the number of patients with PJI is continually increasing^8^. Hitherto, the diagnostic criteria for different PJIs are largely dependent on pathogen detection, and the effectiveness and success of PJI treatment are dependent on the early identification of these pathogens. The traditional method of pathogen detection is the identification of microorganisms with culture which provides a low positive rate^9,10^ and a prolonged duration, causing missed window period for optimal sensitive antibiotic treatment and decreased efficacy. Therefore, early identification of pathogenic microorganisms is essential.

Metagenomic next-generation sequencing (mNGS) is a culture-independent massively parallel DNA sequencing technology and it has become possible to rapidly and comprehensively sequence all of the microbial genetic material within a given sample^11–14^. In 2014, the study on the application of mNGS in the diagnosis of central nervous system leptospirosis infection was first published^15^. Since then, mNGS has been gradually applied to the pathogen detection of various infections, including PJI. The team of Patel reported the process of mNGS based on Illumina platform to detect pathogens of joint fluid and prosthetic sonicate fluid in patients undergoing joint revision, and found that the sensitivity of mNGS was significantly higher than culture and the most existing molecular diagnostic methods^16,17^. But, another study by Kildow et al.^18^ reported that mNGS was not superior to culture in PJI diagnosis. In order to evaluate the diagnostic performance of mNGS for PJI, the intraoperative specimens (deep-tissue and joint fluid) of 95 patients undergoing revision arthroplasty after failed TJR were collected to detect pathogens by culture and mNGS, and the sensitivity and specificity were compared in this study.

## Methods

This is a prospective, single-center, clinical study. Patients who previously had undergone hip and knee replacement undergoing revision due to joint infection, pain and prosthetic loosening from January 2018 to January 2021 in our institution by a single surgeon were prospectively enrolled in this study and signed the informed consent. Patients were excluded if obvious contamination occurred, or without enough joint fluid (< 2 mL) to get tested. Preoperative antibiotics were withheld 2 weeks prior to the surgical procedure until samples were collected. In all cases, serum C-reactive protein (CRP) and erythrocyte sedimentation rate (ESR) were collected and synovial fluid was also assessed for white blood-cell (WBC) count, polymorphonuclear neutrophil (PMN) percentage, leukocyte esterase. This study was approved by the Ethics Committee of Honghui Hospital Affiliated to Xi’an Jiao Tong University (No. 202006004) and performed in accordance with relevant guidelines and regulations.

### Sampling

Synovial fluid and deep-tissue were obtained for all cases at the time of the surgical procedure. Synovial fluid was obtained in a sterile fashion, using an 18-gauge needle prior to arthrotomy. Deep-tissue in at least three different areas were obtained from periprosthetic tissue with the most obvious inflammatory changes and medullary canals. All the samples were equally divided into two, one for culture and another for mNGS. Specimens for culture were promptly partitioned into separate sterile vials. Specimens for mNGS including synovial fluid and deep-tissue were separately partitioned into one special container that was sterile and free of nucleases or other amplification inhibitors and processed by the laboratory within 4 h.

### Culture

Deep-tissue specimens were homogenized and routine culture including aerobic and anaerobic bacterial culture, fungal culture, and acid-fast bacillus culture were proceeded at the same time. Synovial fluid was inoculated half into aerobic and half into anaerobic blood culture flasks (BACTEC 9240 system; BD Diagnostic Systems) and incubated on the BACTEC 9240 instrument (BD Diagnostic Systems). All samples were cultured for 14 days in general. While in the event of a negative result, the culture would be prolonged to 21 days. The culture media with positive results were analyzed to identify the types of bacteria. The culture results of each subject were recorded and the types of bacteria were identified from positive results. In all processes, corresponding negative controls were set up to avoid the adverse effect of contamination.

### mNGS detection

The mNGS workflow included sample preparation, nucleic acid extraction, construction of DNA libraries, metagenomic sequencing and bioinformatics analysis. Full details are available in the Supplemental Methods. DNA extraction of synovial fluid was performed using the TIANamp Micro DNA Kit (DP316, TIANGEN Biotech, China). DNA extraction was performed using the TIANamp Micro DNA Kit (DP316, Tiangen Biotech, China). The construction of DNA libraries was prepared using the MGIEasy FS DNA Library Prep Kit (MGI Tech, China). Sequencing was performed using the BGISEQ-500 platform (BGI-Tianjin, China). A negative control and a positive control of a known pathogen were set up for the same batch of samples. If obvious contamination was found, the specimen would be retest again.

### Definitions

Patients were retrospectively categorized as infected or aseptic using the Musculoskeletal Infection Society (MSIS) criteria^19^ after revision surgery. If any microorganism was detected in synovial fluid or deep-tissue by culture, it was deemed as a positive result; otherwise, it was deemed a negative result. For mNGS, the pathogens were screened according to the predetermined detection threshold after the contamination was eliminated by referring to the negative controls of the same batch. The consistency between positive culture and positive mNGS results in species-level was evaluated. When the pathogens detected by mNGS contained those detected by culture, it was deemed as complete consistency. When the pathogens detected by mNGS did not contain all those detected by culture, it was deemed as partial consistency. When the pathogens detected by mNGS were completely different from those detected by culture, it was deemed as inconsistency. The consistency of the species of pathogen detected by mNGS in the two types of specimen was also evaluated. When the pathogens detected in one of the specimen types were identical or contained by another specimen type, it was deemed as complete consistency. When the pathogens detected in one of the specimen types did not contain all those detected in another specimen type, it was deemed as partial consistency. When the pathogens detected in the two types of specimen were completely different, it was deemed as inconsistency.

### Statistical analysis

A power analysis was conducted to determine the sample size. Using prior institutional data on molecular techniques and aiming for a 30% difference in sensitivity between mNGS and culture, a power of 80%, and an alpha error of 0.05, a sample size of 75 cases was determined.

The sensitivity, specificity, positive and negative predictive values and likelihood ratios of culture and mNGS were determined against the MSIS criteria which in our investigation was considered as the “gold standard”. The weighted Youden index^20^ [jω = 2 (ω × sensitivity +  (1 − ω) × specificity)-1, ω = 0.6] was used to evaluate the diagnostic performance of culture and mNGS for PJI due to unequal importance of sensitivity and specificity. Since we were more concerned with the value of screening suspicious patients (meaning sensitivity) with culture or mNGS then we set ω = 0.6.

Student’s t-test was used to calculate the differences in continuous variables between groups, while chi-squared analysis was used to measure the differences in categorical variables. The sensitivity, specificity, positive and negative predictive values were compared between culture and mNGS using the McNemar test. Rank sum test was used to compare the parameters of mNGS. Statistical significance was defined as p < 0.05 (for a two-sided test). Statistical analyses were performed using SPSS version 17 (IBM Inc., Armonk, NY).

## Results

### General characteristics

Nine cases were excluded from the study due to contamination (1 case), synovial fluid with incomplete diagnostic tests (7 cases) and sample failing to pass the quality control of mNGS (1 case). The remaining 95 cases (39 hips and 56 knees) with 442 samples were included in the study. Overall, 58 cases were categorized as infected (MSIS positive) and 37 were considered aseptic (MSIS negative) (Table 1). The infected and aseptic cohorts did not differ statistically in terms of demographics with no difference in age, gender, body mass index (BMI), and joint location. There was a statistically significant difference for serum CRP, serum ESR, synovial fluid WBC count, and synovial fluid PMN percentage between infected and aseptic cohorts.

**Table 1:** Clinical information of the 95 cases.

Characteristic	MSIS + (N = 58)	MSIS − (N = 37)	p
Age* (years)	67.1 ± 12.3	68.2 ± 12.3	0.71
Gender^**#**^ (male/female)	25/33	17/20	0.62
BMI* (kg/m^2^)	25.1 ± 5.2	23.4 ± 6.1	0.15
Joint			0.59
Hip^†^-initial diagnosis	22 (37.9%)	17 (45.9%)	
Joint infection^**#**^	16	0	
Prosthetic loosening^**#**^	2	15	
Joint pain^**#**^	4	2	
Knee^†^-initial diagnosis	36 (62.1%)	20 (54.1%)	
Joint infection^**#**^	30	0	
Prosthetic loosening^**#**^	3	18	
Joint pain^**#**^	3	2	
MSIS criteria			
Major criteria^†^			
2 positive culture	33 (56.9%)	0 (0.0%)	< 0.001
Sinus tract connecting with prosthesis	15 (25.9%)	0 (0.0%)	< 0.001
Minor criteria			
Serum CRP (mg/L)*	66.8 ± 39.3	6.6 ± 7.3	< 0.001
Serum ESR (mm/h)*	70.8 ± 31.3	21.3 ± 16.9	< 0.001
Synovial fluid WBC count (cells/μL)*	54,855.7 ± 61,989.5	1560.6 ± 2124.5	< 0.001
Synovial fluid PMN percentage (%)*	90.1 ± 12.2	35.6 ± 16.9	< 0.001

*MSIS*+ MSIS positive, *MSIS− *MSIS negative, *BMI* Body mass index, *CRP* C-reactive protein, *WBC* White blood cell, *PMN* Polymorphonuclear neutrophil.

*The values indicate mean and standard deviation.

^#^The values indicate the number of cases.

^†^The values are the number of cases, with the percentage in parentheses.

### Culture and mNGS detection results

A total of 442 samples (95 specimens of synovial fluid and 347 specimens of deep-tissue) were collected for culture. Meanwhile, a total of 190 samples (95 specimens of synovial fluid and 95 specimens of deep-tissue) were collected for mNGS.

**Figure 1:**
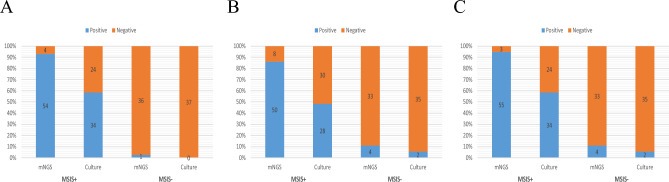
Bar graphs showed the comparison between mNGS and culture results based on MSIS criteria. **(A)** Culture and mNGS results of deep tissue. **(B)** Culture and mNGS results of synovial fluid. **(C)** Culture and mNGS results under the combination of deep tissue and synovial fluid. *mNGS* metagenomic next-generation sequencing, *MSIS*+ MSIS positive, *MSIS− *MSIS negative.

For deep-tissue specimens, culture was positive in 34 infected cases (34/58, 48.3%), and metagenomic next-generation sequencing was positive in 54 infected cases (54/58, 93.1%) and 1 aseptic cases (1/37, 2.7%) (Fig. 1A). For synovial fluid specimens, culture was positive in 28 infected cases (28/58, 58.6%) and 2 aseptic cases (2/37, 5.4%). Metagenomic next-generation sequencing was positive in 50 infected cases (50/58, 86.2%) and 4 aseptic cases (4/37, 10.8%) (Fig. 1B). When the two types of specimen were combined, 36 cases had positive culture results and 59 cases had positive mNGS results. Overall, culture was positive in 34 infected cases (34/58, 58.6%) and 2 aseptic cases (2/37, 5.4%). Metagenomic next-generation sequencing was positive in 55 infected cases (55/58, 94.8%) and 4 aseptic cases (4/37, 10.8%) (Fig. 1C). Poly-organisms were detected by mNGS in 6 infected and 2 aseptic cases (8/59, 13.6%), and 2 species of organism were detected by culture in one aseptic case (1/36, 2.8%).

Among the 36 culture positive cases, 34 infected and 1 aseptic cases had positive mNGS results, and 1 aseptic case had a negative mNGS result (*Staphylococcus aureus* was detected by synovial fluid culture). Among the 34 infected cases with positive culture and positive mNGS results, 33 cases showed complete consistency (33/34, 97.1%), 1 case was inconsistent at species-level while consistent at genus-level (*Streptococcus sanguinis* was detected by deep-tissue culture, while *Streptococcus pneumoniae* and *Streptococcus mitis* were detected by both deep-tissue and synovial fluid mNGS testing), showing an acceptable concordance between mNGS and culture. Among the 33 infected cases with complete consistency of the results, 4 had other potential pathogens detected by mNGS. For the aseptic one with partial consistent culture and mNGS results, *Staphylococcus aureus* and *Staphylococcus epidermidis* were detected by synovial fluid culture, while *Staphylococcus epidermidis* was detected by mNGS testing of synovial fluid.

Among the 59 cases who showed negative culture results, 24 cases were classified as infected (MSIS-positive) and 35 were aseptic (MSIS-negative). Among the 24 infected cases with negative culture results, mNGS was able to identify an organism in 21 cases (21/24, 87.5%), while 2 cases who had no pathogens detected either by mNGS or by culture were diagnosed with infection through the wounded sinus tract, and the remaining 1 patient had an abscess in the joint cavity during replacement and finally diagnosed as infection through a positive pathological diagnosis after replacement. Of the 35 aspetic cases who showed negative culture results, 3 cases had positive mNGS results but low sequence numbers of the detected pathogens.

The mNGS results of deep-tissue and joint fluid were both positive in 49 PJIs. Among them, the species of pathogen detected by mNGS were completely consistent in 43 cases, partially consistent in 5 cases (more pathogens were detected in deep-tissue in 2 cases and more pathogens were detected in joint fluid in 3 cases), and inconsistent in 1 case. Among the aseptic cases, the single one positive sequencing result of deep-tissue was identical with synovial fluid.

The performance of mNGS could be further revealed by major sequencing parameters. An average of 28,420,803 reads (15,133,370–45,274,168) were obtained per sample, among which the mean pathogen sequencing reads was 1615 (1–35,875). There was no significant difference in the total read counts between specimens of deep-tissue and joint fluid (p > 0.05). For PJIs (MSIS+), the mean genome coverage rate of the pathogens which were detected in specimens of deep-tissue (6.5% ± 12.4%) was significantly high than that in synovial fluid (1.8% ± 2.7%; p < 0.001). In general, the higher the genome coverage rates of detected pathogens, the higher the reliability of the detection results. In terms of pathogen sequencing reads, the mean counts in the specimens of deep-tissue (2,010.3 ± 5079.6) was five times more than that in synovial fluid (353.2 ± 1615.2; p < 0.001). Moreover, the read counts were regarded as a semi-quantitative indicator of the detected pathogens. For species abundance, specimens of deep-tissue (44.6% ± 38.6%) showed no significant advantage than synovial fluid (39.2% ± 40.1%; p > 0.05).

### Comparison of sensitivity and specificity between culture and mNGS

The MSIS criteria was considered as the “gold standard”. To diagnose PJI, mNGS outperformed culture in terms of sensitivity, regardless of whether the two types of specimen were combined or not (Table 2). Overall, mNGS demonstrated high sensitivity (94.8%), specificity (89.2%), positive predictive value (93.2%) and negative predictive value (91.7%). In contrast, culture demonstrated high specificity (94.6%) and positive predictive value (94.4%) but low sensitivity (58.6%) and negative predictive value (59.3%). The sensitivity and negative predictive value of mNGS were higher than culture with a difference of 36.2% (p < 0.001) and 32.4% (p = 0.001), respectively, and the specificity (p = 0.617) and positive predictive value (p = 0.813) were similar with no statistically significant difference.

**Table 2:** Sensitivity, specificity, positive and negative predictive values and likelihood ratios for the detection of any bacteria on mNGS compared to organism isolation by culture.

Test methods	Sensitivity* (%)	Specificity* (%)	PPV* (%)	NPV * (%)	PLR*	NLR*	jω
Culture of DT + SF	58.6 (45.0–71.1)	94.6 (80.5–99.1)	94.4 (80.0–99.0)	59.3 (45.8–71.7)	10.8 (2.8–42.5)	0.4 (0.3–0.6)	0.46
Culture of DT	58.6 (45.0–71.1)	100 (88.3–100)	100 (87.4–100)	60.7 (47.3–72.7)	Infinity	0.4 (0.3–0.6)	0.50
Culture of SF	48.3 (35.1–61.7)	94.6 (80.5–99.1)	93.3 (76.5–98.8)	53.8 (41.1–66.1)	8.9 (2.3–35.3)	0.5 (0.4–0.7)	0.34
mNGS of DT + SF	94.8 (84.7–98.7)	89.2 (73.6–96.5)	93.2 (82.7–97.8)	91.7 (76.4–97.8)	8.8 (3.5–22.2)	0.1 (0.02–0.2)	0.85
mNGS of DT	93.1 (82.5–97.8)	97.3 (84.2–99.9)	98.2 (89.0–99.9)	90.0 (75.4–96.7)	34.4 (5.0–238.4)	0.1 (0.02–0.2)	0.90
mNGS of SF	86.2 (74.1–93.4)	89.2 (73.6–96.5)	92.6 (81.3–97.6)	80.5 (64.6–90.6)	8.0 (3.1–20.2)	0.2 (0.1–0.3)	0.75

*mNGS* metagenomic next-generation sequencing, *PPV* positive predictive value, *NPV* negative predictive value, *PLR* positive likelihood ratio, *NLR* negative likelihood ratio, *DT* deep tissue, *SF* synovial fluid.

*The values are given as the estimate, with the 95% confidence interval (CI) in parentheses.

The sensitivity of deep-tissue (93.1%) was close to the combination of the two types of specimen (94.8%) in mNGS, and higher than synovial fluid (86.2%) with no statistically significant difference (p = 0.221), and its sensitivity in culture (58.6%) was significantly higher (p = 0.041) than that of synovial fluid (48.3%). Both the sensitivity of deep-tissue and synovial fluid in mNGS were significantly higher than that of culture, either in combination or solely of the two types of specimen (p < 0.001). The specificity of deep-tissue and synovial fluid in mNGS were close to that of culture, with no statistically significant difference (p > 0.05).

### Comparison of time consumption between culture and mNGS

In the process of mNGS, logistics taken 1–4 h, samples preparation taken 0.5–1 h, nucleic acid extraction taken 2.5–4 h, construction and quantification of DNA library taken 5 ~ 6 h, metagenomic sequencing and bioinformatics analysis taken 12–18 h. It might take a minimum of 21 h to fulfill a report. From sampling to reporting, the average days needed for mNGS (1.3 [95% CI 0.9–1.7]) was significantly shorter than that of culture (5.2 [95% CI 3.1–7.3]). The student’s t-test was used to compare the difference between the two methods (t = 9.878, p < 0.001).

### Composition of organisms

The microbial characteristics detected by mNGS and culture are shown in Table 3. A total of 8 species of organism were detected by culture and 26 by mNGS, among which the most common were *Staphylococcus aureus*, *Staphylococcus epidermidis*, and *Escherichia coli* in sequence. The total detected frequency of corresponding organisms in the specimens of deep-tissue and synovial fluid by mNGS was 59 and 61, respectively. The total detected frequency of corresponding organisms in all specimens by mNGS was 70.

**Table 3:** Correspondence between culture growth and the organisms detected by next-generation sequencing.

Organisms	Culture (N = 36)		mNGS (N = 59)		Culture vs mNGS (DT + SF)^†^
	DT*	SF*	DT*	SF*	
*Staphylococcus aureus*	14	16	14	15	16/15
*Staphylococcus epidermidis*	8	9	8	9	9/9
*Escherichia coli*	5	3	8	7	5/8
*Enterobacter cloacae*	2	1	3	4	2/5
*Enterococcus faecalis*	2	1	2	2	2/3
*Streptococcus sanguinis*	1	0	0	0	1/0
*Klebsiella pneumoniae*	1	0	2	2	1/3
*Candida albicans*	1	1	1	1	1/1
*Pseudomonas aeruginosa*	0	0	4	3	0/4
*Cutibacterium acnes*	0	0	1	2	0/3
*Staphylococcus lentus*	0	0	2	1	0/2
*Streptococcus pneumoniae*	0	0	2	1	0/2
*Serratia marcescens*	0	0	1	2	0/2
*Aspergillus flavus*	0	0	1	0	0/1
*Staphylococcus warneri*	0	0	1	1	0/1
*Staphylococcus capitis*	0	0	1	1	0/1
*Streptococcus lactis*	0	0	1	1	0/1
*Staphylococcus haemolyticus*	0	0	1	1	0/1
*Staphylococcus cohnii*	0	0	1	1	0/1
*Enterobacter hormaechei*	0	0	0	1	0/1
*Finegoldia magna*	0	0	1	1	0/1
*Anaerococcus prevotii*	0	0	1	1	0/1
*Pseudomonas oleovorans*	0	0	0	1	0/1
*Corynebacterium kutscher*	0	0	1	1	0/1
*Streptococcus mitis*	0	0	1	1	0/1
*Mycobacterium tuberculosis*	0	0	1	1	0/1

*mNGS* metagenomic next-generation sequencing, *DT* deep tissue, *SF* synovial fluid.

*The values are given as the detected frequency of a corresponding organism in each single types of specimen.

^†^The values are given as the detected frequency of a corresponding organism in all types of specimen by culture versus the detected frequency of a corresponding organism in all types of specimen by mNGS.

**Figure 2:**
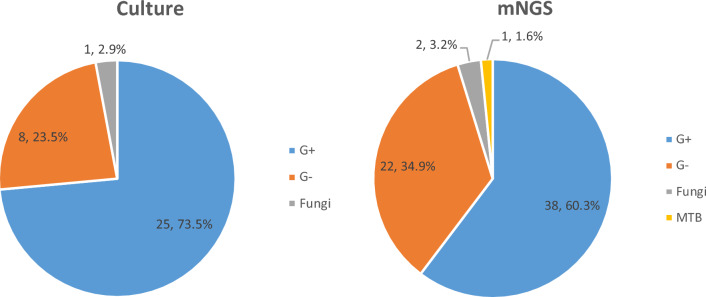
Pie chart showing the proportion of organisms detected by culture and mNGS in all types of specimen in PJIs. *mNGS* metagenomic next-generation sequencing, *MTB Mycobacterium tuberculosis*, *G*+ gram-positive bacteria, *G− *gram-negative bacteria.

For all PJIs, gram-positive bacteria were most frequently detected by culture, followed by gram-negative bacteria and fungi. The similarity is that gram-positive bacteria were most frequently detected by mNGS, followed by gram-negative bacteria, fungi and mycobacterium tuberculosis (Fig. 2).

### Antimicrobial therapy

For all patients with a positive culture, intravenous antimicrobial therapy was initiated to cover pathogens in accordance with culture. For culture-negative PJIs with positive mNGS results and PJIs with microbial culture suggesting monobacterial infection but mNGS suggesting poly-microbial infection and cases with complete inconsistent results obtained from culture and mNGS, three criteria were used to determine whether the mNGS results were “true positives” (see Supplemental Methods). If the mNGS results were “true positives”, our infectious disease physicians would consider the findings and tailor the antimicrobial therapy on the basis of mNGS results. For culture-negative PJIs with negative mNGS results, empirical therapy which was the combination of intravenous quinolones and vancomycin with oral rifampicin was administered. The antimicrobial therapy would be adjusted according infection control and the the suggestion of infectious disease physicians. For aseptic patients with positive mNGS results, more frequent follow-ups were conducted.

## Discussion

Periprosthetic joint infection is a catastrophic complication after total joint replacement, but its accurate diagnosis and treatment are challenging for clinicians^21^. Either in the diagnosis of PJI according to the diagnostic criteria introduced by the Musculoskeletal Infection Society (MSIS), the Infectious Diseases Society of America (IDSA), or the European Bone and Joint Infection Society (EBJIS) or in the treatment of PJI by debridement, antibiotics, and implant retention (DAIR) or one-stage or two-stage revision, the key is to identify the pathogens. Otherwise, it is difficult to determine the antibiotics, which in turn leads to poor efficacy, drug tolerance, delayed treatment, and severe complications.

The culture result is still the gold standard for pathogen detection^22^. However, due to the wide variety of microorganisms, harsh conditions for the growth of some pathogenic bacteria, limitations of the traditional culture technique, formation of the bacterial biofilm, and use of antibiotics before culture, the culture has a low positive rate. Therefore, multiple periprosthetic tissue specimens should be submitted for cultures to improve the positive detection rate. In a study of 40 cases with PJI, Fink et al.^23^ considered that deep-tissue culture was significantly more sensitive than synovial fluid culture in the diagnosis of infection after knee arthroplasty. However, a study involving 110 cases with infection after joint replacement conducted by Cross et al.^24^ reported that deep-tissue culture had no advantage in the detection of organism comparing with synovial fluid culture. In this study, the sensitivity of deep-tissue culture was 58.6%, which was significantly higher than that of synovial fluid culture. Compared with 2 false positives in synovial fluid culture, there were none in deep-tissue culture, although no statistically difference were found in specificity between the two types of specimen. In our opinion, culture of deep-tissue is more recommended. It is reported that the culture with ultrasonic cracking fluids prepared by the ultrasonic oscillation of the prosthesis of PJIs could have a higher positive rate but still less than 70%^25–27^, and although it had a higher sensitivity than the culture with tissues or arthrocentesis fluids, it might cause false-positive results due to contamination, according to Prieto-Borja et al.^28^. Furthermore, culture does not have obvious advantage for rapid diagnosis. Therefore, a method to identify pathogens timely and accurately is imperative in clinical practice.

Metagenomic next-generation sequencing, emerging in recent years, is a high-quality laboratory technique for diagnosis. It identifies the species of pathogens by acquiring sequence information and aligning to the microbial database. Theoretically, it enables comprehensive and unbiased detection of all rare and atypical pathogenic organisms in samples and the judgment of infections by other pathogens; also, it is highly sensitive and accurate and can be accomplished in a short duration^29^. However, similar to the culture, antibiotics should be discontinued 2 weeks before mNGS detection because the sequence number might decrease after antibiotic therapy, which would reduce the sensitivity^30^. A total of 95 cases including 58 cases categorized as infected and 37 cases categorized as aseptic according to the MSIS criteria were recruited in this study. Among these 95 cases, 36 cases had positive culture results and 59 cases had positive mNGS results. Culture was positive in 34 infected cases (58.6%) and 2 aseptic cases (5.4%). Metagenomic next-generation sequencing was positive in 55 infected cases (94.8%) and 4 aseptic cases (10.8%). Among the 24 cases diagnosed with infection who had negative culture results, 21 harbored potential pathogens detected by mNGS with a rate of 87.5%, demonstrating utility in difficult-to-diagnose infections. It could widely facilitate the determination of reasonable and effective postoperative antibiotic treatment. Because mNGS is more sensitive to detected pathogens than culture, many potential pathogens were detected by mNGS in aseptic cases. Even so, the specificity of mNGS was comparable to culture and its ability to screen infections were significantly better than culture according to the weighted Youden index which was 0.85 and 0.64, respectively. Thoendel et al.^17^ reported the results of 408 sonicate fluid samples, including culture-positive PJIs, culture-negative PJIs, and aseptic failures, were tested by culture and mNGS. Comparing with sonicate fluid culture, mNGS identified known pathogens in 94.8% of culture-positive infections and detected potential pathogens in 43.9% of culture-negative infections. Detection of organisms in samples from uninfected aseptic failure cases was conversely rare (3.6%). Several studies reported that the positive detection rate of mNGS in PJIs was 87.5–92.5%^31–33^ and that in the samples having negative culture results was 81.8%^29^, similar to the results of the present study.

To diagnose PJI, the combination of deep-tissue culture and synovial fluid culture demonstrated high specificity (94.6%) and positive predictive value (94.4%) but low sensitivity (58.6%) and negative predictive value (59.3%). In contrast, mNGS demonstrated high sensitivity (94.8%), specificity (89.2%), positive predictive value (93.2%) and negative predictive value (91.7%). The sensitivity and negative predictive value of mNGS were higher than culture with a difference of 36.2% and 32.4%, respectively, and the specificity positive predictive value were similar with no statistically significant difference. This suggested that, compared with culture, mNGS could identify pathogens as much as possible to help diagnose infection and avoid misdiagnosis and missed diagnosis, and further guide clinical treatment. Meanwhile, the results were a little bit different after differentiation between the sample types was performed. The sensitivity of deep-tissue (93.1%) higher than synovial fluid (86.2%) with no statistically difference, and its sensitivity in culture (58.6%) was significantly higher than that of synovial fluid (48.3%). The specificity of deep-tissue and synovial fluid in mNGS were close to that of culture, with no statistically significant difference. Compared with 4 false positives in synovial fluid sequencing and 2 in synovial fluid culture, there were 1 false positive in deep-tissue sequencing and none in deep-tissue culture, although no statistically difference were found in specificity between the two types of specimen. Multiple deep-tissue specimens showed more advantages than synovial fluid in detection of pathogens. Cai et al.^34^ included 44 cases who were suspected of PJI and underwent surgery to detect pathogens by mNGS and culture. The sensitivity, specificity and accuracy of periprosthetic tissue mNGS in the diagnosis of PJI were 95.45%, 90.91% and 93.18%, respectively. However, in a study of He et al.^31^, 177 specimens including periprosthetic tissues, synovial fluid, and prosthetic sonicate fluid were collected to detect pathogens by culture and mNGS. The overall sensitivity and specificity of mNGS in the diagnosis of PJI were 95% and 94.7%, respectively. Among the three types of specimen, the sensitivity of periprosthetic tissues was the lowest. In general, the sampling site of periprosthetic tissue significantly affected the quantity of organisms. In addition, compared to synovial fluid, tissue significantly increased the proportion of human factors, resulting in less proportion of microbial reads, which may lead to a decrease in the sensitivity of mNGS. Therefore, homogenization of the tissues and degradation of human DNA before DNA extraction may significantly improve the positive detection rate of mNGS. In this study, deep-tissue were processed as described above to obtain high sensitivity.

In this study, pathogens were detected in 4 aseptic cases by mNGS, and 1 of them was also culture positive. The false positives may be due to the possibility of non-pathogenic organisms in presumed aseptic revision cases. Interestingly, one presumed aseptic case with *Pseudomonas oleovorans* detected by mNGS later developed recurrent PJI and *Pseudomonas oleovorans* was detected again using mNGS technique. According to the literature, pathogens could be detected around aseptic loosening prosthesis and the prevalence of unexpected positive culture was 9.2%, which indicated the possibility of subclinical infection^35^. It was also reported that partial unrecognized or occult infection should be implicated in failure of prosthetic joints believed to be aseptic^16^. In addition, there have been articles published talking about positivity of mNGS in normal synovial fluid in patients who were not infected^36^. This so-called synovial fluid biome exists in which nonpathogenic organisms have been found in normal synovial fluid. Moreover, partial organisms detected by mNGS in aseptic samples were opportunistic pathogens, such as skin colonization bacteria, which might infiltrate the deep wound during surgical procedures and result in false positives. In theory, all microbial molecular diagnostic methods faced the interference of background microorganisms, and so did mNGS. However, parameters of mNGS including read counts, abundance and genome coverage rate could be simultaneously analyzed to help distinguish background contaminant reads from bacteria present in the samples^37^. Contamination could also eliminated by referring to the same batch of negative control. Therefore, a reasonable threshold was set to reasonably interpret the mNGS results to balance the sensitivity and specificity. In addition, the severity of clinical symptoms, CRP, ESR and other molecular markers should be taken into consideration.

Among the 34 culture-positive PJIs, 33 cases (33/34, 97.1%) showed complete consistency of the results between culture and mNGS, 1 case was inconsistent at species-level while consistent at genus-level. Among the 33 cases with complete consistency of the results, 4 had other potential pathogens detected by mNGS, of which 1–2 were the main pathogens. The mNGS results of deep-tissue and joint fluid were both positive in 49 PJIs. Among them, the species of pathogen detected by mNGS were completely consistent in 43 cases, partially consistent in 5 cases and completely inconsistent in 1 case. The high concordance showing an acceptable reliability and stability of mNGS. Tarabichi et al.^38^ collected 86 joint fluid samples using culture and mNGS to detect suspected infections, and the consistency rate of pathogen detection results between the two methods was 96.1%. Huang et al.^39^ evaluated the concordance characteristic of the pathogens detected by mNGS and culture and concordance between different types of sample, which was similar to the present study. For patients with positive nNGS and positive culture results, the matched and partly matched cases made up to 91.7% (11/12). For PJIs with positive mNGS results of sonicate fluid and joint fluid, the matched and partly matched cases made up to 94.4% (17/18).

Among the 58 cases who were categorized as infected (MSIS-positive) in the present study, mNGS could not detect pathogens in 3 cases. The appearance of false positives possibly caused by the following reasons: First, target DNA might be degraded before sequencing due to improper preservation of specimens or unstandardized procedure^40^; Second, the detection of target sequences was affected by large amounts of human DNA remaining in the samples; Third, whether 2 weeks of discontinuity of antibiotics is sufficient before detection as reported previously is yet to be elucidated, and the antibiotic treatment might reduce the sequence number of pathogenic bacteria in samples during mNGS detection leading to a negative result; Fourth, the insufficient amount of target pathogens in sample was too difficult to distinguish from background organisms. In addition, considering that the cultures of these 3 cases were also negative, the negative mNGS results may be caused by improper sampling sites and methods, and the possibility of misdiagnosis according to MSIS criteria could not be ruled out, after all, the specificity of MSIS criteria is not up to 100% for now.

The days needed from sampling to reporting of culture and mNGS was also recorded in this study. The average time needed for culture was 5.2 days, and 1.3 days for mNGS, indicating that mNGS detection required a significantly shorter duration than the traditional culture, which was more conducive to the early targeted antibiotic treatment for PJIs. According to a study by Torchia et al.^41^, mNGS detection was more cost-effective than the traditional culture, and hence, should be used as the standard method in PJIs in clinical practice.

Furthermore, a total of 8 species of pathogen were detected by culture and 26 by mNGS, and 5 cases diagnosed with infection had other potential pathogens detected by mNGS. A major reason for the failure of PJI treatment was that the pathogenic microorganisms might be infected by other or multiple pathogenic bacteria^42,43^. The failure of media to simulate the growth environments of different microorganisms imposes some limitations on the culture, while mNGS detection does not require any media and can detect fungi and *Mycobacterium tuberculosis* that are difficult to be detected by culture. These features overcome the effects of the competitive growth of bacteria in the culture under traditional conditions and multiple infections.

In conclusion, mNGS had a significantly higher rate than culture in PJI detection but required a short duration, rendering it conducive to the early diagnosis and accurate treatment of PJI, and hence, should be widely applied. However, the traditional culture can guide the use of antibiotics through drug sensitivity test, while mNGS detection, although can detect the drug-resistant genes of pathogenic bacteria^44^, fails to define the expression of such drug-resistant genes; thus, its role to guide is limited and requires additional exploration. In addition, mNGS detection is also defective because its results might be false-positive or false-negative and fails to distinguish between infected and contaminated organisms.

Nevertheless, the present study has some limitations: First, it had a small sample size, which might affect the reliability of the conclusion. Second, it was a single-center study, which might be subject to some biases. In the next stage, multicenter, randomized-controlled, and prospective studies with a larger sample size should be carried out, patients included in the studies should be followed up, and the recurrence of infections should be considered for accurate and reliable conclusions.

## Supplementary Information

Supplementary Information 1.

Supplementary Information 2.

## Data Availability

The datasets generated and/or analysed during the current study are available in the Sequence Read Archive (SRA) repository, [PRJNA877476].
