# Supplementary material for: Direct detection and identification of periprosthetic joint infection pathogens by metagenomic next-generation sequencing
Source: Sci Rep. 2023 May 16;13:7897. doi: 10.1038/s41598-023-35215-3 (PMC10188433; doi:10.1038/s41598-023-35215-3)
Supplement: Supplementary file 1 — Supplementary Information 1. [file 41598_2023_35215_MOESM1_ESM.pdf]

Direct Detection and Identification of Periprosthetic Joint Infection Pathogens by Metagenomic Next-generation Sequencing

Linjie Hao, Pengfei Wen, Wei Song, Binfei Zhang, Yanjie Wu, Yumin Zhang, Tao Ma &Yusheng Qiu

Sample preparation and DNA exaction

During pretreatment, deep-tissue specimens were homogenized through the Qiagen TissueLyser II instrument (Qiagen). Then, 1.5-mL microcentrifuge tubes with 0.5 mL of sample (deep-tissue homogenate or synovial fluid) and 1 g of 0.5-mm glass beads were attached to a horizontal platform on a vortex mixer and agitated vigorously at 2800–3200 rpm for 30 min. After agitation, 0.3 mL of the sample was separated into a new 1.5-mL microcentrifuge tube, and DNA extraction was performed using the TIANamp Micro DNA Kit (DP316, Tiangen Biotech, China) according to the manufacturer's recommendation. Add Proteinase K to the microcentrifuge tube and mix thoroughly. Then add Buffer GB to the sample, mix thoroughly, and incubate at 56°C for 10 min to yield a homogeneous solution. Briefly centrifuge the 1.5 ml microcentrifuge tube to remove drops from the inside of the lid. Add ethanol (96-100%) to the sample. Mix thoroughly and Incubate at room temperature for 5 minutes. Briefly centrifuge the 1.5 ml microcentrifuge tube to remove drops from the inside of the lid. Pipet the mixture from step 4 into the Spin Column CR2 (in a 2 ml Collection Tube) and centrifuge at 12,000 rpm (~13,400 × g) for 30 sec. Discard flow-through and place the spin column into the Collection Tube. Add Buffer GD (Ensure ethanol has been added) to Spin Column CR2, and centrifuge at 12,000 rpm (~13,400 × g) for 30 sec. Then discard the flow-through and place the spin column into the Collection Tube. Add Buffer PW (Ensure ethanol has been added) to Spin Column CR2, and centrifuge at 12,000 rpm (~13,400 × g) for 30 sec. Discard the flow-through and place the spin column into the Collection Tube. Add Buffer PW (Ensure ethanol has been added) to Spin Column CR2 again, and centrifuge at 12,000 rpm (~13,400 × g) for 30 sec. Discard the flow-through and place the spin column into the Collection Tube. Centrifuge at 12,000 rpm (~13,400 × g) for 2 min to dry the membrane completely. Place the Spin Column CR2 in a new clean 1.5 ml microcentrifuge tube, and pipet Buffer TB directly to the center of the membrane. Incubate at room temperature for 2-5 min, and then centrifuge for 2 min at 12,000 rpm (~13,400 × g). The extracted DNA should be stored at -20°C.

Construction of DNA libraries

The construction of DNA library was prepared using the MGIEasy FS DNA Library Prep Kit (MGI Tech., China) through an end-repair method. By randomly breaking template DNA into fragments (200~300bp) through Covaris S220 (Thermo Fisher Science, USA) ultrasonic crusher. End Repair and A-tailing were performed simultaneously. Add MGIEasy DNA Adapters and purify the adapter-ligated DNA with beads. After polymerase chain reaction (PCR) amplification was performed, purification of the PCR product was conducted again. The quality of the DNA libraries was assessed using an Agilent 2100 Bioanalyzer (Agilent Technologies, Santa Clara, California) combined with quantitative PCR to measure the adapters before sequencing.

Metagenomic sequencing and bioinformatics analysis

The qualified DNA library was denatured and cyclified to form a single strand circularization. After the process of rolling circle amplification, DNA nanoball was prepared and loaded onto the Patterned Array. Sequencing was performed using the BGISEQ-500 platform. A negative control and a positive control of a known pathogen were set up for the same batch of samples. If obvious contamination was found, the specimen was retested again. The bioinformatics analysis included the following main steps: 1) High-quality sequencing data were generated by removing short (shorter than 35 bp), low-quality, low-complexity reads, adapter contamination, and duplicated reads. 2) Human host sequences were eliminated by mapping to the human reference genome (hg19) with Burrows-Wheeler Alignment (http://bio-bwa.sourceforge.net). 3) The remaining sequencing data were aligned to the current bacterial, virus, and fungal databases (ftp://ftp.ncbi.nlm.nih.gov/genomes/), which contain the genomic sequences of 4,061 viruses, 2,473 bacteria and 199 fungi related to human diseases. The reference genomes in the database were downloaded from the National Center for Biotechnology Information (NCBI). The contamination was eliminated by referring to the same batch of negative control. Reference genome coverage was also used to help distinguish background contaminant reads from bacteria present in the sample. Pathogens were ranked according to the read counts, genome coverage rate and species abundance and screened according to detection threshold.

Interpretation of mNGS results (Figure 1)

The sequencing algorithm we adopted in this study with reference to the analysis method by Ivy MI et al[1].

1.Bacteria (except Mycobacterium)

If the genome coverage rates of organisms were 10 times or more than that of other organisms, they were considered positive. If not, and the organisms were not the same contaminant bacteria in negative control, in the meanwhile, reads⩾10, they were considered positive. If the organisms belonged to the contaminant bacteria in the negative control, it will be retested. If the result was still the same as the previous, when the genome coverage rate⩾2% and reads⩾10, they were considered positive. If the organisms were not belonged to contaminant bacteria in negative control and genus reads⩾10, they were positive at the genus-level.

2.Fungi and virus

Due to the low biomass of these organisms in DNA extraction, if reads⩾10 in genus-level or species-level, and the read counts were in the top 10 of bacteria, they were considered positive.

3.Mycobacterium:

Due to the low biomass of these organisms in DNA extraction, if reads⩾1 and the read counts were in the top 10 of the overall bacteria, they were considered positive.

4.Parasites were not considered to be pathogens in general.

5. *Burkholderia, Ralstonia, Delftia, Sphingobium, Alternaria, Sodaria, Aspergillus, Albugo*, and other genera were the most common background bacteria and were also detected in other sample types in our laboratory.

6. For cases with negative microbial culture but positive mNGS results and cases with

microbial culture suggesting monobacterial infections but mNGS suggesting multiple infections and cases with complete inconsistent results obtained from culture and mNGS, three criteria were used to determine whether the mNGS results were "true positives" based on previous literature[2,3]: (1) using a third method, the results were consistent with the results of mNGS, such as 16S PCR. (2) Pathogens were clearly reported in osteoarticular infection, according to previous studies, which was consistent with the clinical characteristics of the patient. (3) Targeted treatment response determined by at least three senior clinicians.

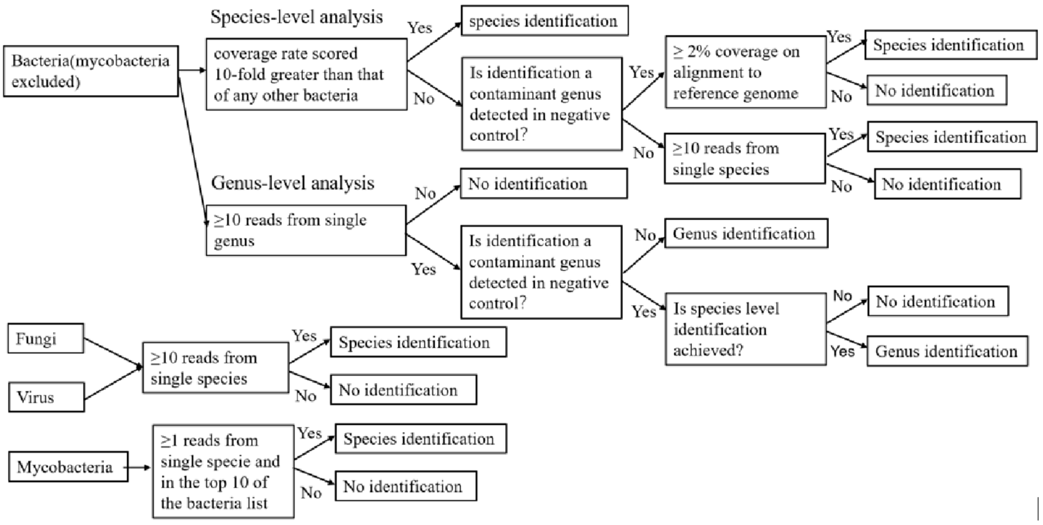

Figure 1. The identification algorithm of a positive mNGS results

Reference

[1] Ivy, M. I. et al. Direct Detection and Identification of Prosthetic Joint Infection Pathogens in Synovial Fluid by Metagenomic Shotgun Sequencing. *J Clin Microbiol.* 56(9), e00402-18 (2018).

[2] Wang J. et al. Metagenomic next-generation sequencing for mixed pulmonary infection diagnosis. *BMC Pulm Med.* 19(1), 252 (2019).

[3] Xie G. et al. Exploring the clinical utility of metagenomic next-generation sequencing in the diagnosis of pulmonary infection. *Infect Dis Ther.* 10(3):1419–1435 (2021).
